# Editorial: Novel therapeutic approaches for the treatment of ocular disease, Volume II

**DOI:** 10.3389/fphar.2022.1104105

**Published:** 2022-12-01

**Authors:** Kyriaki Thermos, Stephanie C. Joachim

**Affiliations:** ^1^ Department of Pharmacology, School of Medicine, University of Crete, Heraklion, Greece; ^2^ Experimental Eye Research Institute, University Eye Hospital, Ruhr-University Bochum, Bochum, Germany

**Keywords:** ocular disease, oxidative stress, neurodegeration, neuroinflammation, neovasuclarization, therapeutics

The Research Topic “Novel Therapeutic Approaches for the Treatment of Ocular Disease, Volume II” provides information related to ocular disease pathophysiology and therapeutics and also presents a paradigm of the use of deep learning (DL)-based technique to identify risk factors and create a prediction model for disease, such as neovascular age-related macular degeneration (nAMD). Age-related cataract, AMD, diabetic retinopathy, and glaucoma are leading causes of visual impairment and loss of vision. Key factors in the pathogenesis of eye disease include oxidative stress, neurodegeneration, neuroinflammation, and neovascularization.

Age-related cataract (ARC), opacification of the lens, is the leading cause of blindness worldwide. Surgical treatment is currently the only effective option for cataract management. Oxidative stress and apoptosis of the human lens epithelial cells (HLECs) is believed to be the cause of cataract formation. Yang et al. employed Na_2_SeO_3_ to establish a model of *in vivo* cataract and H_2_O_2_ to simulate HLECs oxidative injury *in vitro* with the aim to investigate the efficacy of a pentacyclic triterpene, acetyl-11-keto-β-boswellic acid (AKBA), as an antioxidant and antiapoptotic, and the putative mechanism involved in cataract formation. Their findings suggested that AKBA alleviated oxidative injury and cataract formation *via* the activation of the Keap1/Nrf2/HO-1 signal (Yang et al.).

Retinal pigment epithelial (RPE) cells maintain the functional integrity of photoreceptors and choroid acting as selective barriers. Ocular disorders such as diabetic retinopathy, AMD and fibrotic retinal disorders are a result of oxidative stress induced pathological/dysfunctional RPE. Park et al. investigated the mechanism of action of the bioactive flavonol, fisetin, as an antioxidant in human RPE cells (ARPE-19 cell line). Fisetin afforded ROS scavenging activity *via* a mechanism involving Nrf2-mediated activation of the HO-1 enzyme. These findings reinforce the tenet that Nrf2/HO-1 cascade is a putative target for the discovery of new therapeutics for ocular disease (Park et al.).

The human ARPE-19 cell line was also employed to examine the role of transforming growth factor-beta (TGF-β1) in the regulation of epithelial mesenchymal transition (EMT) migration and the downstream mechanisms involved in tumor metastasis and suppression. EMT contributes to various fibrotic retinal disorders. Lee et al., in search of a therapeutic target for fibrotic retinal disorders, examined the effect to the recombinant human cluster of differentiation 82 (*rh*CD82), a tumor metastasis suppressor that represses the functions of motility related proteins to restrain cell migration and invasion on TGF-βinduced EMT. It was reported that *rh*CD82 suppressed TGF-β1-induced EMT *via* a mechanism involving the blockade of smad-dependent signaling in ARPE-19 cells. These findings recommend CD82 as a potential therapeutic target in fibrotic retinal disorders (Lee et al.).

An interesting pathway leading to the development of putative new therapeutics for neovascular AMD is presented in the study by El-Darzi et al. Three different players are involved in this study, namely the cholesterol metabolic enzyme**,** CYP46A1 (cytochrome P450 46A1), 5XFAD mice, a model of rapid amyloidogenesis in Alzheimer’s disease (AD), and the anti-HIV drug efavirenz (EFV). 5XFAD mice develop subretinal and RPE deposits associated with retinal vascular lesions. Treatment with a small dose of EFV increased the activity of CYP46A1 and enhanced retinal cholesterol turnover. The treated mice had a reduction in deposit size and vascular lesion frequency along with the leakage on fluorescein angiography. The size of focal accumulations of Aβ plaques, unesterified cholesterol, and Oil Red O-positive lipids were also reduced, as was the activation of retinal macrophages/microglial cells. The authors suggest, after similar studies in brain, that EFV should be safe to be investigated in patients with neovascular AMD (El-Darzi et al.). This will be a good strategy of drug repositioning which is becoming a promising field in drug discovery.

Liu et al. aimed to analyze the response of microglia in the oxygen induced retinopathy (OIR) mouse (C57BL/6J) model examining the time course of retinal microglial status, ramified (resting) vs. amoeboid (activated), and their quantity. Immunohistochemisty colocalization studies, using markers for microglia and retinal vessels, were carried out in whole-mounted retinae (P12 to P30) at different retinal locations of physiological (room air) and pathological (OIR) mouse retinas. Confocal microscopy images were obtained and microglia and vessel relationship analyses were carried out to compare the differences between the physiological and OIR groups. They report that microglia in superficial and deep retinal areas displayed different patterns in response to retinal vascular changes. Activated microglia were observed even after the retinal vasculature appeared normal. In addition, pharmacological inhibition of microglia during both hyperoxic and hypoxic stages of OIR led to exacerbated vascular damage. The authors suggest that retinal microglia might play a protective role in retinal vascular maintenance during the OIR process. They also state that targeting microglial activation may prevent and treat ROP at early and late stages (Liu et al.).

Song et al. applied deep learning (DL)-based techniques to analyze pre-therapeutic and post-therapeutic spectral domain optical coherence tomography (SD-OCT) images from patients with neovascular age-related macular degeneration (nAMD). The idea was to identify imaging biomarkers in these patients and develop a model to predict persistent disease activity after anti-VEGF treatment. Especially information about the ellipsoid zone (EZ) and the external limiting membrane (ELM) proved the performance of the model. This model had sensitivity of 0.920 and a specificity of 0.962 to predict 1-year disease activity and could be useful for an image-guided prediction of long-term disease nAMD patients (Song et al.).

In summary, the original articles in this Research Topic cover different retinal and ocular diseases. Findings from this Research Topic could contribute to a better understanding of disease pathogenesis, deep learning-based techniques for diagnostics, as well as novel treatment options.

